# Combined Transcriptome Sequencing of *Mycoplasma hyopneumoniae* and Infected Pig Lung Tissue Reveals Up-Regulation of Bacterial F1-Like ATPase and Down-Regulation of the P102 Cilium Adhesin *in vivo*

**DOI:** 10.3389/fmicb.2020.01679

**Published:** 2020-07-20

**Authors:** Tjerko Kamminga, Nirupama Benis, Vitor Martins dos Santos, Jetta J. E. Bijlsma, Peter J. Schaap

**Affiliations:** ^1^Laboratory of Systems and Synthetic Biology, Department of Agrotechnology and Food Sciences, Wageningen University and Research, Wageningen, Netherlands; ^2^Bioprocess Technology and Support, MSD Animal Health, Boxmeer, Netherlands; ^3^Discovery and Technology, MSD Animal Health, Boxmeer, Netherlands

**Keywords:** *Mycoplasma hyopneumoniae*, RNA sequencing, pathogen enrichment, infection, host-pathogen interaction, F1-like ATPase, P102 cilium adhesin

## Abstract

*Mycoplasma hyopneumoniae* (*M. hyopneumoniae*) causes enzootic pneumonia in pigs but it is still largely unknown which host-pathogen interactions enable persistent infection and cause disease. In this study, we analyzed the host and bacterial transcriptomes during infection using RNA sequencing. Comparison of the transcriptome of lung lesion tissue from infected pigs with lung tissue from non-infected animals, identified 424 differentially expressed genes (FDR < 0.01 and fold change > 1.5LOG2). These genes were part of the following major pathways of the immune system: interleukin signaling (type 4, 10, 13, and 18), regulation of Toll-like receptors by endogenous ligand and activation of C3 and C5 in the complement system. Besides analyzing the lung transcriptome, a sampling protocol was developed to obtain enough bacterial mRNA from infected lung tissue for RNA sequencing. This was done by flushing infected lobes in the lung, and subsequently enriching for bacterial RNA. On average, 2.2 million bacterial reads were obtained per biological replicate to analyze the bacterial *in vivo* transcriptome. We compared the *in vivo* bacterial transcriptome with the transcriptome of bacteria grown *in vitro* and identified 22 up-regulated and 30 down-regulated genes (FDR < 0.01 and fold change > 2LOG2). Six out of seven genes in the operon encoding the mycoplasma specific F1-like ATPase (MHP_RS02445-MHP_RS02475) and all genes in the operon MHP_RS01965-MHP_RS01990 with functions related to nucleotide metabolism, spermidine transport and glycerol-3-phoshate transport were up-regulated *in vivo*. Down-regulated *in vivo* were genes related to glycerol uptake, cilium adhesion (P102), cell division and myo-inositol metabolism. In addition to providing a novel method to isolate bacterial mRNA from infected lung, this study provided insights into changes in gene expression during infection, which could help development of novel treatment strategies against enzootic pneumonia caused by *M. hyopneumoniae*.

## Introduction

*Mycoplasma hyopneumoniae* causes enzootic pneumonia in pigs (Maes et al., 1996), a mild, chronic pneumonia characterized by a non-productive, dry cough. Infected pigs often develop secondary infections which makes *M. hyopneumoniae* an important contributor to the development of respiratory disease complex in pigs (Brockmeier et al., 2002) and a major threat to the worldwide pig industry. The pathogen easily spreads within and between herd populations via nose-to-nose contact and aerosols. Treatment with antibiotics results in a decrease in symptoms but not in eradication of the disease. Vaccination with adjuvanted inactivated bacterial vaccines is effective to control disease symptoms but does not prevent colonization of the lung. Improvement of housing conditions and herd management practices can also decrease disease prevalence (Maes et al., 2008). There is a need for improved treatment or prevention methods but development of these methods is hampered because the exact mechanisms used by *M. hyopneumoniae* to colonize and survive in the pig lung are not completely known (Liu et al., 2013; Bin et al., 2014).

Investigations into *M. hyopneumoniae*-host interactions using histological techniques have shown that this pathogen specifically colonizes the lower respiratory tract by binding to cilia on the epithelium of trachea, bronchi and bronchioles. The entire length of the cilia can be bound by bacteria but interaction with the epithelial cell body is rare (Tajima and Yagihashi, 1982; Blanchard et al., 1992; DeBey and Ross, 1994). During colonization, cilia can be degraded and bacterial cells can form multilayered structures resembling biofilms (Raymond et al., 2018). Infected lungs often show tissue inflammation, visible as lung lesions. It has been established that the production of cytokines: interleukin-1 (IL-1α and β), IL-2, IL-4, IL-6, IL-8, IL-10, IL-12, IL-18, and tumor necrosis factor alpha (TNF-alpha) occurs during *M. hyopneumoniae* infection (Rodríguez et al., 2004; Thanawongnuwech et al., 2004; Choi et al., 2006; Muneta et al., 2006). Vaccinated animals showed increased levels of *M. hyopneumoniae* specific antibodies and an altered cell-mediated immune response after challenge, indicating that both a local mucosal immune response as well as a cell-mediated immune response are required to reduce symptoms caused by *M. hyopneumoniae* infection (Thacker et al., 2000).

*M. hyopneumoniae* is not known to be motile and does not form motility structures, such as those found in *M. mobile* and *M. pneumoniae*, yet the bacterium successfully evades the protective action of the mucociliary escalator. *M. hyopneumoniae* expresses a large diversity of proteolytically-cleaved multifunctional cilium adhesion proteins on the bacterial cell surface, which bind glycosaminoglycans (e.g., heparin, fibronectin and plasminogen) (Djordjevic et al., 2004; Deutscher et al., 2010; Seymour et al., 2010, 2012). Most abundantly present on the cell surface are fragments from P97 and P102 proteins for which genes are present in paralogous gene families with six copies per gene in the *M. hyopneumoniae* strain 232 genome (Minion et al., 2004). Many of these paralogs are present in two gene transcriptional units with one copy per gene and were found to be expressed *in vivo* (Adams et al., 2005). Besides the P97/P102 paralogous families, two other *M. hyopneumoniae* genes function as cilium adhesins: P159 (MHP_RS02535) which is proteolytically cleaved into three fragments that bind heparin (Raymond et al., 2013) and M42 glutamyl aminopeptidase (MHP_RS01270) which binds heparin and plasminogen (Robinson et al., 2013). The repertoire of proteins used for adhesion is probably even more extensive since many intracellular proteins were also found to be present on the cell surface suggesting a possible role in adhesion (Reolon et al., 2014).

When attached to the ciliated epithelium, multiple bacterial lipases, proteases and nucleases could release nutrients for growth but specific virulence factors have not been described. Multiple lipoproteins (P65, P50, P44, and P70) are expressed at the bacterial cell surface and were found to be highly immunogenic. P65 (MHP_RS03425) was found to be a lipolytic enzyme with a preference for short-chain fatty acids (Schmidt et al., 2004). Upstream of the P65 gene lies a region with tandem repeats which is expected to cause slippage of DNA polymerase which could cause variation in the expression of the P65 protein (Vasconcelos et al., 2005; Ferreira and De Castro, 2007). P65 has two paralogs in the *M. hyopneumoniae* strain 232 genome (MHP_RS02755 and MHP_RS00345) (Minion et al., 2004). Functions for the other lipoproteins are unknown.

The recruitment of plasminogen and activation to plasmin, facilitated by M42 glutamyl aminopeptidase and leucine aminopeptidase, are potential mechanisms that could cause tissue damage (Lähteenmäki et al., 2005; Robinson et al., 2013; Jarocki et al., 2015). Further damage to host tissue could be caused by the production of oxidizing compounds, such as hydrogen peroxide and hydrogen sulfide, as was reported for other mycoplasma species (Brennan and Feinstein, 1969; Vilei and Frey, 2001; Schmidl et al., 2011; Großhennig et al., 2015) but whether this mechanism plays a role in *M. hyopneumoniae* infections remains to be established. In *Mycoplasma mycoides* subspecies *capri* proteins have been identified that bind and cleave host IgG which could be a method applied for immune evasion (Arfi et al., 2016). Homologous proteins were identified in the *M. hyopneumoniae* genome but it is not known if this system plays a role during *M. hyopneumoniae* infection. Finally, differences in the metabolic capabilities between commensal and pathogenic swine mycoplasma species have been elucidated using genome-scale metabolic models (Ferrarini et al., 2016). In that study the glycerol pathway, related to hydrogen peroxide production and the myo-inositol pathway, uniquely present in *M. hyopneumoniae* when compared to other mycoplasma species, were reported as possible pathways related to virulence.

Better understanding of the role of the adhesive proteins, possible virulence factors and metabolic pathways in the bacteria and the response of the host to infection could be obtained by sequencing of the *in vivo* transcriptomes. Gene expression in *M. hyopneumoniae* during infection was studied *in vivo* using microarrays by Madsen et al. (2008). In that study, glycerol metabolism was found to be up-regulated *in vivo* supporting the possible role of glycerol oxidase during infection. Determination of the transcriptome of pathogens with microarrays has several disadvantages, such as: probe sequence bias, non-specific hybridization and limitations in the dynamic range in expression levels that can be measured (Croucher and Thomson, 2010). Because of these disadvantages, microarrays have been widely replaced by RNA-sequencing.

Studying bacterial gene expression during infection with RNA sequencing is challenging because the ratio of host RNA to bacterial RNA is very high in infected tissue which makes it hard to obtain enough bacterial reads to study bacterial expression levels. So far, only one study analyzed *in vivo* gene expression of a mycoplasma species using RNA sequencing (Pflaum et al., 2015) but that study did not give a global insight in the transcriptional landscape of the bacterium inside the host.

To identify novel vaccine candidates or treatment strategies there is a need for detailed understanding of the interactions between *M. hyopneumoniae* and the host. In this study we aimed to perform dual mRNA sequencing of *M. hyopneumoniae* infections using whole tissue and lung flush RNA extracts. However, from whole tissue, not enough bacterial mRNA could be extracted. Thus, to assess bacterial transcriptional changes during infection, we developed a sampling method to isolate sufficient quantities of bacterial RNA from lung flushes and compared the transcriptomes of *M. hyopneumoniae* during *in vivo* growth with culture grown (*in vitro*) cells. In this study unique insight was gained in the bacterial *in vivo* transcriptome which could be used to further develop novel vaccine candidates or treatment strategies.

## Materials and Methods

### Sequencing and *in vitro* Cultivation of *M. hyopneumoniae* Strain 98

*Mycoplasma hyopneumoniae* strain 98, a Danish field isolate strain (provided by Dr. N. Friis, National Veterinary Laboratory, Copenhagen) was used. The strain was grown in 100 ml FRIIS medium (Friis, 1975) in 250 ml closed glass bottles at 37°C with agitation (100 RPM). Cultures were sampled during exponential growth indicated by an increase in titer measured using flow cytometry (FACSMicroCount, BD). Mycoplasma cells were pelleted using centrifugation (3 min at 9000 g’s) and the cell pellet was directly frozen (<-15°C) for DNA extraction or submerged in RNA later (Ambion) and stored at 2–8°C for RNA extraction. DNA extraction for genome sequencing from the pelleted frozen cells was done using the Gentra Puregene bacterial kit (Qiagen). Genome sequencing was done using Illumina HiSeq2500 (paired-end, 500 MB, 250 bp read length). Genome assembly was done using the Ray algorithm (Boisvert et al., 2010). Annotation of the genome was done using SAPP (Koehorst et al., 2016) in which Prodigal v. 2.6.2 (Hyatt et al., 2010) was used for gene calling and InterProScan v.5.17-56.0 (Jones et al., 2014) for protein domain annotation. A reciprocal best-blast hit analysis (Wolf and Koonin, 2012) was done using BLAST + (Camacho et al., 2009) to find orthologous proteins in *M. hyopneumoniae* strain 232 (Minion et al., 2004), filtering for matches with a minimum of 70% identity and 50% query coverage. For interpretation of the differentially expressed bacterial genes we mention the locus tags of genes present in strain 232 found with the BLAST + analysis. NCBI reference genomes used to compare the protein domain repertoire were: NC_006360 (strain 232), NC_007295 (strain J), NC_007332 (strain 7448), NC_017509.1 (strain 168), NC_021283 (strain 168L), and NC_021831 (strain 7422). For strain 11 accession MWWN00000000 was used. The genome sequence of strain 98 is available from the NCBI under accession number WTQC00000000.

### Animal Studies

Animal studies were performed after approval by an ethical commission and according to national regulations in The Netherlands. In total thirteen pigs were used for this study from three animal trials performed at MSD Animal Health. Nine healthy pigs from a *M. hyopneumoniae* free herd were challenged intra-tracheally on two consecutive days with 10 ml of *M. hyopneumoniae* strain 98 culture containing ± 10^7^ CCU/ml. Pigs were anesthetized, euthanized and exsanguinated 3 weeks after challenge. Within a short time-period (<5 min) after the necropsy the lungs were removed and tissue samples were obtained and directly submerged in RNA later (Ambion) or infected lobes were flushed with RNA later using a method previously described (Mengeling et al., 1995) but adapted for this specific purpose. Briefly, an infected lobe was selected for sampling based on the presence of lesions in the lobe. A plastic pipette (7 ml total volume) was used to dispense 5 ml of RNA later (Ambion) into the bronchus toward the selected lobe. Directly after dispensing, the fluid was retrieved from the lobe and stored at 2–8°C for further processing. Four healthy pigs were necropsied unchallenged at 7 weeks ± 3 days of age.

### RNA Extraction

RNA extraction from tissue samples was done using the RNeasy mini kit (Qiagen) following the manufacturer’s protocol. Lesions in infected lungs were identified by discoloration of tissue and from the affected tissue, total RNA was extracted for sequencing. RNA extraction from flush samples (0.75 ml) was done with 7.5 ml of Trizol LS reagent (Ambion) following the manufacturer’s protocol. For bacterial pellets, RNA later was removed and 3.75 ml Trizol LS reagent was added. RNA was precipitated using glycogen as co-precipitant as described in the Trizol extraction protocol. After RNA re-suspension, the quantity was determined using NanoDrop. RNA samples were treated with Turbo DNase (Ambion) to remove potential DNA contamination following the manufacturer’s protocol. Enrichment of bacterial RNA in the flush samples and culture samples was done using the MICROBEnrich kit (Ambion) and the quality of all samples was determined using Experion RNA StdSense (Bio-Rad).

### RNA Sequencing and Read Mapping

Kallisto (Bray et al., 2016) was used to determine estimated counts on gene transcripts using pseudoalignment to a pig reference transcript file from Ensembl (all cDNA version 11.1, FTP location)^1^. To analyze the read distribution of prokaryotic reads on the *M. hyopneumoniae* strain 98 genome, rRNA was removed using the Ribo-Zero Gold rRNA Removal Kit (Epidemiology). rRNA-depleted RNA was fragmented to an average length of 100–200 base pairs and converted to double-stranded complementary DNA (cDNA). Strand specific library preparation was done using a protocol based on the “dUTP (deoxyuridine triphosphate) method.” The Illumina stranded TruSeq RNA-seq library preparation kit was used. Sequencing of the library was done using the Illumina HiSeq 2500: single-end reads, 50 cycles. Quality assessment of reads was done as previously described (Kamminga et al., 2017b). Reads were aligned to the strain 98 genome using TopHat v2.1.0 (Kim et al., 2013) with the standard settings for strand-specific alignment enabled (fr-firststrand). Accepted hits found by TopHat were sorted using samtools v1.3.1 (Li et al., 2009), from the sorted hits a BED-file was created using bamToBed and coverage per gene was calculated using coverageBed, both programs from the bedtools suite v2.17.0 (Quinlan and Hall, 2010). Reads mapping to non-coding RNAs, were determined using the same methods after creation of a ncRNA annotation file for the strain 98 genome based on the best blast hit (Camacho et al., 2009) with the annotated ncRNAs in strain 232 (Lloréns-Rico et al., 2016). Raw data files used for this study are available from the NCBI bioproject PRJNA593525.

### Analysis of Differentially Expressed Genes and Differentially Expressed ncRNAs

The R package edgeR (Robinson et al., 2009; McCarthy et al., 2012) was used for analysis of differentially expressed genes and differentially expressed ncRNAs (for simplicity we refer to both as genes) between the *in vitro* and *in vivo* condition. Only genes with a read count of >100 counts per million (CPM) in two or more datasets were kept as described by Rienksma et al. (2015). Data was normalized for RNA composition by calculating scale factors (based on the trimmed mean of *M*-values; Robinson and Oshlack, 2010) which were used to correct the library size. Common dispersion was calculated based on conditional maximum likelihood, correcting for library size differences based on pseudocounts estimated per quantile, first by assuming a poisson distribution to calculate the common estimated dispersion. This initially calculated common dispersion was used to get a final estimate for the pseudocounts and a final estimate of the common dispersion (Robinson and Smyth, 2008). Two types of variation contribute to the total variance in the probability distribution: technical and biological variation. Biological variation (BCV) represents the variation between biological replicates that would remain if sequencing depth is infinite. Dispersion per gene (biological variation) was calculated using an empirical Bayes estimation with weighted likelihood (Robinson and Smyth, 2007). Differential expression was calculated for each gene using a pairwise exact testing method taking into account the tag-wise dispersion estimates. False discovery rates were controlled using the algorithm by Benjamini and Hochberg (1995). Genes with an FDR < 0.01 and a LOG2 fold change larger than 2 were considered to be significantly different. For eukaryotic genes an FDR threshold of 0.01 and fold change threshold of 1.5 was used to check for significance. A more conservative cut-off value for the fold change of differentially expressed bacterial genes was used because low RNA quantities were obtained from infected lung flushes.

## Results and Discussion

### Sequencing of *M. hyopneumoniae* Strain 98 Revealed No Novel Protein Domains

Genome assembly using the filtered Illumina FastQ sequence reads (average Phred quality score: 36.17) resulted in a draft genome sequence of 19 scaffolds. The total genome size was 880.620 bp and the GC% was 28.5%. We compared the protein domain content of strain 98 to the domain content of other *Mycoplasma hyopneumoniae* strains (232, J, 11, 7448, 7422, 168, and 168L, Supplementary Table S1) obtained after genome annotation using SAPP (Koehorst et al., 2016; Kamminga et al., 2017a). We found a total core domainome size of 843 unique protein domains and an accessory domainome of 23 protein domains (Supplementary Table S2). With a reciprocal-best blast hit analysis 601 orthologous proteins were identified in strain 98 when compared to strain 232. Based on the composition of the accessory domainome, strain 98 is mostly related to *M. hyopneumoniae* strain 7448 (Supplementary Figure S1). Overall, the differences were small and no novel protein domains were identified in the strain 98 genome.

### RNAseq of Infected Lung Tissue Showed Upregulation of Inflammatory Response Pathways

**FIGURE 1:**
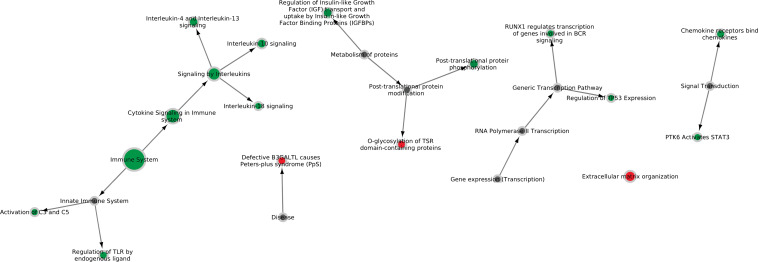
Six major Reactome pathways found differentially expressed in lung tissue infected with *M. hyopneumoniae*. Nodes are colored green if the majority of genes in the pathway are up-regulated *in vivo* and red if the majority of genes in the pathway are down-regulated *in vivo*. The size of the nodes reflects the number of genes significantly expressed in the pathway. Gray nodes are root notes or nodes that connect to root nodes (non-specific pathways without gene information).

Comparison of RNA sequencing datasets of three infected tissue samples with four non-infected tissue samples showed 424 genes that were significantly differentially expressed in the infected animals versus the controls (Supplementary Table S3). Reactome pathways containing these 424 genes (filtered for significance based on *p* < 0.01) were analyzed for overrepresentation of differentially expressed genes. In total 17 pathways were enriched (Table 1), related to interleukin signaling, detection of chemokines, tissue damage (regulation TLR, O-fucosylation = Peters syndrome, TSR), STAT3 activation, B cell receptor (BCR) signaling and complement activation (Figure 1). Specifically, interleukin-10, interleukin-4, interleukin-13, and interleukin-18 related pathways were found up-regulated. Increased expression of pro-inflammatory cytokines was expected in infected lung tissue and increased levels of IL-4, IL-10, and IL-18 were previously determined using immunohistochemical and ELISA methods (Rodríguez et al., 2004; Thanawongnuwech et al., 2004; Choi et al., 2006; Muneta et al., 2006). Increased expression of IL-13 was not previously described during *M. hyopneumoniae* infection and should be verified by measuring protein levels. We did not identify upregulation of all interleukins previously found upregulated during infection (IL-1, IL-2, IL-6, IL-8, IL-12) or of TNF-alpha production. This could be because we used a different analysis method, had animals with a different genetic background or because we specifically sequenced a lesion where infection is already causing tissue damage.

**TABLE 1:** Differentially expressed pathways in pig lung tissue infected with *M. hyopneumoniae*.

Pathway name	Entities found	Entities total	Entities *p*-value
Interleukin-10 signaling	25	86	3.77E-15
Signaling by interleukins	57	640	5.29E-10
Cytokine signaling in immune system	70	1051	5.74E-07
Interleukin-4 and Interleukin-13 signaling	22	211	1.26E-05
Chemokine receptors bind chemokines	10	48	1.29E-05
Immune system	125	2638	0.0005
Interleukin-18 signaling	4	11	0.0007
Regulation of TLR by endogenous ligand	6	31	0.001
Activation of C3 and C5	3	7	0.002
PTK6 Activates STAT3	3	7	0.002
RUNX1 regulates transcription of genes involved in BCR signaling	3	7	0.002
Post-translational protein phosphorylation	11	109	0.002
Regulation of insulin-like growth factor (IGF) transport and uptake by insulin-like growth factor binding proteins (IGFBPs)	12	127	0.002
Defective B3GALTL causes Peters-plus syndrome (PpS)	6	39	0.003
O-glycosylation of TSR domain-containing proteins	6	41	0.004
Extracellular matrix organization	21	329	0.009
Regulation of TP53 Expression	2	4	0.009

**Differentially expressed genes assigned based on FDR < 0.01 and fold change > 1.5 LOG2. Pathway information was derived from the Reactome database.**

The pathway for regulation of Toll-like receptors by endogenous ligands was upregulated in infected lung tissue. Endogenous ligands are formed when tissue damage occurs. Specific genes identified were TLR1, TLR2, TLR6, LBP, and SFTPA1. A role for TLR2 and TLR6 was described previously (Muneta et al., 2003; Sun et al., 2013) and upregulation of these genes is caused by the presence of lipoproteins on the cell membrane of *M. hyopneumoniae*. A possible upregulation of lipopolysaccharide binding protein (LBP) was not previously described and needs to be further investigated. LBP might be upregulated because glycolipids are present on the *M. hyopneumoniae* membrane and/or because oxidized phospholipids are present that interact with LBP (Erridge and Spickett, 2013). SFTPA1 produces pulmonary surfactant-associated protein A1 which could play a role in killing of *Mycoplasma* by alveolar macrophages (Hickman-Davis et al., 1999). Note that because only tissue from lesions was sequenced by RNAseq, it was not possible to distinguish the immune response to exogenous compounds from the response to endogenous compounds.

### Upregulation of the Complement System (C3 and C5) and B Cell Receptor Signaling

Whether the complement system plays a role in protection against *M. hyopneumoniae* is not known. However, pulmonary alveolar type II epithelial cells have been shown to produce complement proteins in the lung (Pandya and Wilkes, 2014). We identified an increased expression of the C3 and C5 components of the complement system which could be caused by an increased concentration of pro-inflammatory cytokines in the lung. The role of the complement system during *M. hyopneumoniae* infection should be further investigated.

We found upregulation of a pathway which plays a role in B cell receptor signaling and B cell development (RUNX1 regulates transcription of genes involved in BCR signaling). Increased expression in pathways related to B cell receptor signaling is expected when there is a serological response to infection. In previous studies, elevated TNF levels have been measured in infected lungs which could stimulate activation of B cells (Thacker et al., 2000; Thanawongnuwech and Young, 2001). At the moment of necropsy, seroconversion was found for all *M. hyopneumoniae* challenged pigs using the IDEXX *M. hyo* Ab test.

### Dual RNAseq of Whole Lung Tissue Does Not Result in Sufficient Bacterial Reads

Lung tissue samples were obtained from infected animals and sequenced with high capacity. Here, 0.1% of the reads were found to match with the *M. hyopneumoniae* genome (Supplementary Table S4). As a rule of thumb, we assumed that sufficient coverage of a bacterial genome for analysis of gene expression levels is obtained with ≥ 1 million non-rRNA bacterial reads (Westermann et al., 2012). Since insufficient numbers of bacterial reads were obtained, dual RNASeq was considered not economically feasible for this sample type.

**FIGURE 2:**
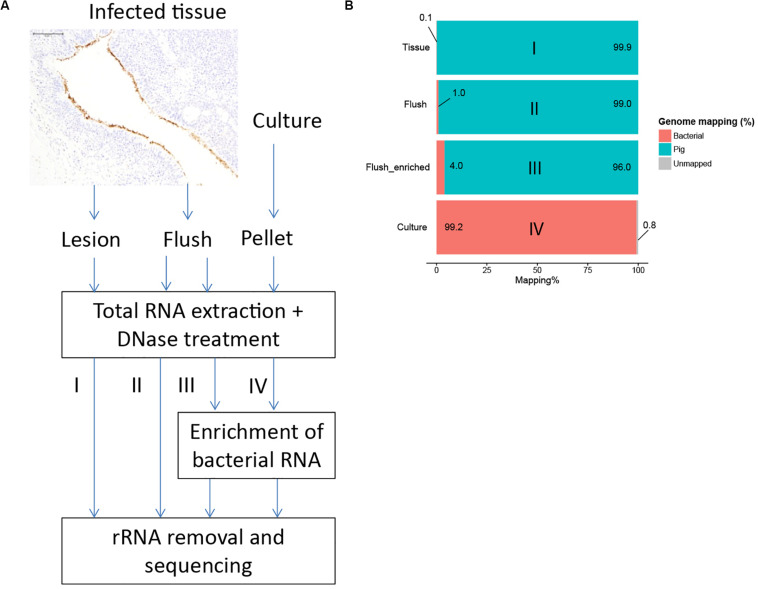
Development of an enrichment strategy to obtain bacterial RNA reads from infected tissue. **(A)** Immunohistochemistry showed that *M. hyopneumoniae* (stained brown) is bound to the respiratory epithelium. Three alternative strategies were tested; I: total RNA extraction from infected tissue; II: RNA extraction from lung flush; III: RNA extraction from lung flush with bacterial enrichment step, IV: *in vitro* control. **(B)** Percentage of reads mapped to the *M. hyopneumoniae* genome.

To overcome this hurdle, we developed alternative methods to isolate bacterial RNA from lungs infected with *M. hyopneumoniae* (Figure 2A). Application of differential lysis techniques to isolate bacterial RNA (Rienksma et al., 2015; Robbe-Saule et al., 2017), was not an option for *Mycoplasma hyopneumoniae* since this bacterium does not contain a cell wall. As previously reported, we identified with immunohistochemistry that *M. hyopneumoniae* is found specifically on the surface of the respiratory epithelium (Figure 2A). To isolate sufficient amounts of bacteria, we decided to flush an infected lung lobe with RNA later and isolate total RNA from the lung flush. Again, we tested if this would be a feasible method to perform dual RNASeq but from non-infected lung flush samples insufficient host RNA concentrations were isolated. We hypothesized that low cell numbers were present at the epithelial surface of non-infected lungs where infected lungs contained a higher cell number as a result of infection. Since we no longer pursued dual RNAseq, an enrichment step for bacterial RNA was implemented in which pig mRNA was removed based on the presence of a poly-A tail (Figure 2A).

The average RNA quantity isolated from infected lung flush samples was low: 4.2 μg (range: 0.17–18.51 μg). Due to the low quantity of RNA it was not possible to assess the RNA quality based on the ratio between 23S and 16S rRNA (Supplementary Figures S2–S6). Therefore, we analyzed the quality of the *in vivo* data with multivariate data analysis. A ten times higher read mapping percentage was achieved by sequencing RNA from a flush sample and even higher read mapping percentages were reached by applying the enrichment step (Figure 2B and Supplementary Table S4). To assess if the enrichment step influenced the expression levels for bacterial genes, we sequenced a flush sample from the same pig with and without applying the enrichment step, all other treatment steps were done in parallel. There was a strong positive correlation between the expression levels in the non-enriched sample compared to the enriched sample (Pearson coefficient = 0.97, Supplementary Figure S7) which showed that the enrichment step and the repeat of the other purification steps did not influence the bacterial read distribution.

We further analyzed the variation in the read distribution per bacterial gene in the various sample types with principal component analysis (PCA) based on the read counts per gene. This analysis showed a separation of *in vitro* samples from *in vivo* samples and again showed that enrichment for bacterial RNA did not influence the general expression landscape in biological replicates (Supplementary Figure S8). This analysis also showed that 69% of the variance in the dataset followed from the sample origin and only 19% variance was found between *in vivo* samples, showing high consistency for both sample types. The method we developed enabled sequencing of bacterial mRNA from infected lung tissue with sufficient read numbers.

A disadvantage of the lung flush method is that the exact location where the bacterial cells resided cannot be determined. We consider it likely that by applying shear force when flushing the lung, cells were removed that were both unbound and bound to the cilia, however an exact ratio could not be determined. It is also not clear if multilayered cell structures (biofilms) were removed by flushing or mainly single cells not bound to the cilia. However, since biofilms were present on the lung surface where cilia were degraded (Raymond et al., 2018), we consider it likely that by flushing both loose cells as well as biofilm structures can be removed for sequencing. The PCA method did not show a major variation between flush samples indicating that, although we do not know exactly the location of the bacterial cells, the lung flush method can be used to consistently obtain bacterial culture from multiple lobes in the pig lung.

### Highly Expressed Bacterial Genes Show Similar Expression Levels *in vivo* and *in vitro*

We analyzed the distribution of sense and antisense mapping reads per gene and found on average 97.5 ± 1.2% sense mapping reads (Supplementary Table S5). Most genes needed for cilium adhesion were highly expressed during infection (Table 2). This was expected because the bacterium specifically binds the ciliated epithelium (Figure 2A; Zielinski et al., 1990; Blanchard et al., 1992; Young et al., 2000). The same genes were also highly expressed *in vitro* which may suggest constitutive expression. Surprisingly, the gene encoding the cilium adhesin P102 (17_1, MHP_RS00920) was repressed during infection (see below). Genes related to cell division and transport, specifically a subunit of the ascorbate transporter and MFS transporter, were also found highly expressed in both conditions. Key metabolic genes found highly expressed under both conditions were related to the final stages of glycolysis (pyruvate dehydrogenase and lactate dehydrogenase) and myo-inositol metabolism. This indicates an important role for these metabolic pathways under both growth conditions. Our results agreed well with data reported by Siqueira et al. (2014) of highly expressed genes in *M. hyopneumoniae* strain 7448 determined with RNA sequencing.

**TABLE 2:** Highly expressed genes in *M. hyopneumoniae in vivo* compared to highly expressed genes *in vitro.*

No.^a^	Gene^b^	Strain 232 locus tag^c^	Average expression flush (RPKM)^d^	Average expression culture (RPKM)^e^	No. culture^f^	Strain 232 functional annotation^g^
1	3_47	MHP_RS02535	493776.2	299689.9	4	P97 paralog (P216)
2	2_38	MHP_RS02100	247739.6	734005.1	1	Ribosomal RNA small subunit methyltransferase H
3	2_39	MHP_RS02105	225848.4	592086.0	2	Cell division protein FtsZ
4	2_37	MHP_RS02095	114276.6	331599.7	3	transcriptional regulator MraZ
5	6_38	MHP_RS01240	109536.7	78273.3	6	MFS transporter
6	6_39	MHP_RS01235	100349.3	79257.0	5	L-lactate dehydrogenase
7	3_48	MHP_RS02540	44267.5	18049.1	10	P159 adhesin
8	7_1	MHP_RS01335	38659.0	22183.2	7	2-oxoisovalerate dehydrogenase subunit beta
9	7_2	MHP_RS01340	37934.2	21524.6	8	Pyruvate dehydrogenase (acetyl-transferring) E1 component subunit alpha
10	6_90	MHP_RS03425	37502.7	4180.8	39	Surface lipoprotein, P65, lipolytic enzyme
11	6_96	MHP_RS03455	36751.8	15342.2	14	Cilium adhesin (P102 paralog)
12	6_97	MHP_RS03460	25312.6	6862.2	30	Cilium adhesin (P146)
13	4_84	MHP_RS00925	24586.3	15767.1	13	Protein p97; cilium adhesin
14	3_1	MHP_RS02285	24475.0	15250.0	15	Hypothetical protein
15	2_21	MHP_RS02015	22410.3	15206.3	16	PTS ascorbate transporter subunit IIC
16	13_7	MHP_RS02625	21366.1	12687.3	18	Hypothetical protein
17	2_22	MHP_RS02020	21133.6	13236.9	17	Phosphotriesterase
18	18_10	MHP_RS00760	20245.6	15971.7	12	3D-(3,5/4)-trihydroxycyclohexane-1,2-dione acylhydrolase (decyclizing)
19	4_83	MHP_RS01395	15054.7	8547.7	23	Hypothetical protein
20	18_6	MHP_RS00780	14894.6	17666.8	11	Methylmalonate-semialdehyde dehydrogenase

**^*a*^Rank of genes in in vivo transcriptome based on expression level (RPKM). ^*b*^Locus tag M. hyopneumoniae strain 98. ^*c*^Locus tag homologous gene M. hyopneumoniae strain 232. ^*d*^Average expression level in flush samples based on read numbers taking into account gene length and total amount of reads in the dataset. ^*e*^Average expression level in culture samples based on read numbers taking into account gene length and total amount of reads in the dataset. ^*f*^Rank of genes in in vitro transcriptome based on expression level (RPKM). ^*g*^Annotated function in strain 232.**

The overlap observed in highly expressed genes could be partly explained by the choice of the cultivation medium. In our study Friis medium was used for *in vitro* cultivation of *M. hyopneumoniae*. This medium contains animal serum, animal derived peptones and yeast extract. The composition of the growth medium will influence the *in vitro* transcriptome of *M. hyopneumoniae*. Since there are components in serum that will also be present in the lung when tissue damage occurs, bacteria could encounter similar components in the lung as in the medium which could result in similar expression levels for certain genes. If a medium was chosen with less animal-derived components, more differences could be observed between *in vitro* and *in vivo* growth, but such a medium is not readily available.

### 53 Bacterial Genes Were Found Differentially Expressed During Infection

**FIGURE 3:**
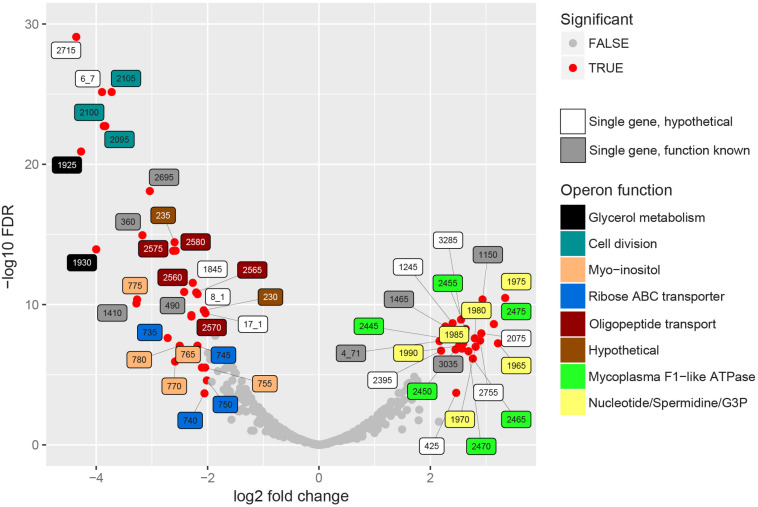
*In vivo* differentially expressed bacterial genes. EdgeR was used to find differentially expressed genes in the RNA seq. datasets of enriched cultures and enriched lung flush samples. Significance in this graph is based on FDR < 0.01 and LOG2 fold change threshold > 2. Significantly up- or down regulated genes are indicated in red, label color shows operon function. If a homologous gene was found in strain 232, the abbreviated locus tag of that gene was used as label number. If no homologous gene was found the strain 98 gene ID was used. Single genes, indicated in white and gray, were either not present in an operon or the only differentially expressed gene in the operon. Supplementary Table S6 shows the complete EdgeR analysis results.

Sense mapping reads were used to analyze differentially expressed genes after filtering for genes with a read count of >100 counts per million (CPM) in two or more datasets (Rienksma et al., 2015). By filtering we removed 203 genes (29% of genes in the genome). Based on FDR < 0.01 and LOG2 fold change larger than two, we found 23 genes up-regulated *in vivo*, 30 genes down-regulated *in vivo* and 445 genes not differentially expressed (Figure 3 and Supplementary Table S6). The average biological coefficient of variance (BCV) calculated for all genes in the dataset using EdgeR was 0.30, meaning that on average 30% of the variation in gene abundance is the result of biological variation between biological replicates. This value for BCV is higher than expected for experiments with genetically identical organisms (McCarthy et al., 2012). The higher variation is possibly the result of varying conditions in the lung, the PCA analysis showed that the majority of the variation within samples types is present in the *in vivo* datasets (Supplementary Figure S8).

#### *In vivo* Up-Regulation of F_1_-Like ATPase in *Mycoplasma hyopneumoniae*

**FIGURE 4:**
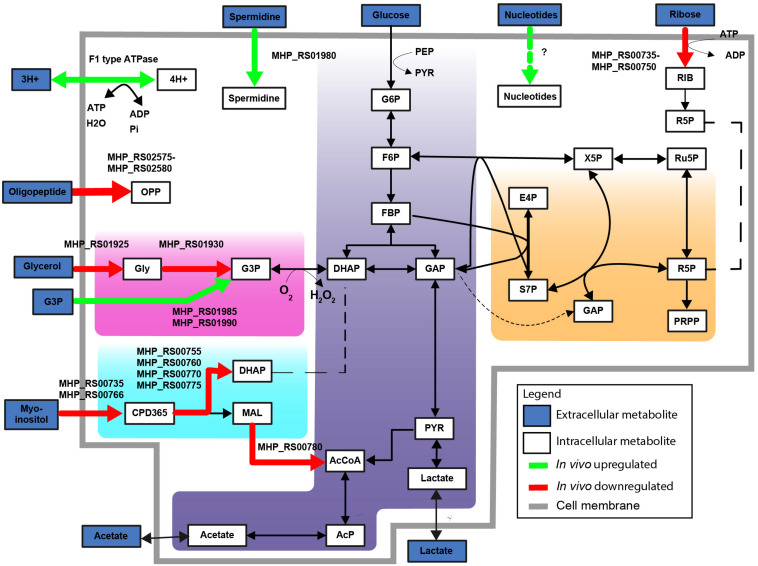
F1 type ATPase and glycerol-3-phosphate transport upregulated *in vivo*. Differentially expressed metabolic genes are shown, up- and downregulated genes are shown based on FDR < 0.01 and LOG2 fold change threshold > 2. Upregulated metabolic genes *in vivo* (green). Downregulated metabolic genes *in vivo* (red). Highlighted simplified pathways are glycolysis (blue), pentose phosphate pathway (orange), glycerol (pink) and myo-inositol metabolism (turquoise). Abbreviations are explained in Supplementary Table S7.

Genes encoding the alpha and beta subunits (MHP_RS02445 and MHP_RS02450) of F_1_-like ATPase and four hypothetical genes in this operon were up-regulated *in vivo* (Figure 3). Interestingly, these genes were not part of the typical operon encoding Type 1 F_1_F_0_ATPase but were part of the operon presumably encoding a Type 3 F_1_-like ATPase (Béven et al., 2012). Based on structural analysis (Béven et al., 2012), one of the hypothetical genes, MHP_RS02465, possibly resembles the γ-subunit of the F_1_-like ATPase. Read levels for the gene that resembles the ε-subunit (MHP_RS02460) were lower than the threshold set for significance and therefore this gene was removed from the dataset. Three other hypothetical proteins (homologs of MHP_RS02455 and MHP_RS02470-MHP_RS02475) also have a possible role in formation of F_1_-like ATPase, two were predicted to be present in the cell membrane, but their exact role remains to be elucidated. There is no differential expression of genes in the operon encoding the typical Type 1 F_1_F_0_ATPase (MHP_RS00240-MHP_RS00280). The role of the F_1_-like ATPase, which originated in the Hominis group of mycoplasma species (Béven et al., 2012) in infection is unclear but might provide protection against pH stress since the ATPase is able to transport protons across the plasma membrane (Figure 4). To form a functional ATPase, components from the F1-like ATPase could possibly interact with components from the standard ATPase. Alternatively, the F1-like ATPase components might have a role in translocation (Béven et al., 2012) but *M. hyopneumoniae* is not known to be motile.

#### Increased Expression of Bacterial Nucleases During Infection

MHP_RS01975, a putative lipoprotein expressed on the cell surface which functions as a calcium dependent exonuclease (Schmidt et al., 2007) was induced *in vivo* (Figure 3). Exonuclease activity could be important to supply nucleotides needed for growth in the host since pathways to synthesize nucleotides *de novo* are not present in mycoplasma species. The role of nucleases in host-pathogen interaction was investigated in multiple mycoplasma species: nucleases from *M. hyorhinis* caused apoptotic symptoms in a human pancreatic adenocarcinoma cell line (Paddenberg et al., 1998), endonuclease P40 expressed by *M. penetrans* had a cytotoxic effect on lymphocytic cell lines (Bendjennat et al., 1999) and MAG_5040 of *M. agalactiae*, which shows sequence similarity with MHP_RS01975, was shown to degrade the DNA component of neutrophil extracellular traps (NETs) (Cacciotto et al., 2016). It was recently shown that *M. hyopneumoniae* can incorporate nucleotides from macrophage extracellular traps (METs) (Henthorn et al., 2018).

We also found up-regulation of the A subunit of excinuclease ABC (uvrABC) involved in DNA repair (MHP_RS01465) and of DNA polymerase I (MHP_RS03035). This could indicate that DNA damage is occurring more frequently under *in vivo* conditions for instance due to oxidative stress induced by neutrophils. Based on our transcriptome analysis of infected lung tissue we would expect neutrophil activity, but we cannot conclude on specific activity of neutrophils on the surface of the lung epithelium. We also found up-regulation of MHP_RS01150, a ribosome small subunit-dependent GTPase, but a possible role during infection for this protein is unclear.

#### *In vivo* Up-Regulation of Bacterial Genes Related to Glycerol-3-Phosphate Transport and Spermidine Transport

Other genes present in the MHP_RS01975 operon were also up-regulated. MHP_RS01985 and MHP_RS01990 were annotated as putative glycerol-3-phosphate transport system permeases (ugpA and ugpE) (Figure 4). Glycerol-3-phosphate could be used as an alternative carbon source (Figure 4) or for cardiolipin biosynthesis. Also present in this operon is MHP_RS01980 (*potA*) which is a potential spermidine/putrescine transporter. Finally, two up-regulated genes in the operon were putative lipoproteins: MHP_RS01965 and MHP_RS01970, with an unknown function and no further functional information could be derived from the protein domain composition of these proteins.

#### P102 Cilium Adhesin Is Down-Regulated During Infection in *Mycoplasma hyopneumoniae*

The gene encoding the cilium adhesin P102 (17_1, MHP_RS00920) was repressed during infection. Adhesion to the cilia is important for *M. hyopneumoniae* to reside on the respiratory epithelium. We also identified down-regulation of P97 cilium adhesin (FDR < 0.01) but with a less than 2 LOG2 fold change (Supplementary Table S6). As indicated the exact location of the bacterial cells obtained by flushing is not known so there could be multiple reasons why genes related to cilium adhesion were downregulated. Possibly, the bacterial RNA was isolated from bacteria already adhered to the ciliated epithelium, this could indicate a down-regulation upon contact with cilia. Alternatively, transcription could be up-regulated in the *in vitro* culture when cells were grown in suspension. Another possible explanation is that by flushing lung lobes we selected for bacteria that were not attached to the cilia resulting in isolation of bacteria with lowered expression of P102.

#### Bacterial Genes Related to Cell Division Were Down-Regulated During Infection

The operon with genes related to cell division (MraZ, RsmH, and ftsZ) was down-regulated *in vivo* (Figure 3). This could indicate a lower growth rate *in vivo* when compared to *in vitro* growth in complex medium. Alternatively, part of the bacterial population isolated by flushing could be in the stationary phase (or even starvation phase) instead of growth phase as these genes were found to be down-regulated in *M. pneumoniae* cultures when entering stationary phase (Güell et al., 2009). We also found down-regulation of MHP_RS00360, chaperone DnaK (70 kDa heat-shock protein) and chaperone protein ClpB (MHP_RS01410). DnaK binds to denatured proteins during heat-shock and ClpB in general binds misfolded and aggregated proteins. Our culture conditions were not at high temperature, possibly these proteins play a role during normal growth and are down-regulated in relation to the growth rate. Genes MHP_RS00490, “MurR/RpiR family transcriptional regulator” and a Type IV secretory system conjugative DNA transfer protein, “ICEF-IIA” (MHP_RS02695) were also found down-regulated but the role of these genes in *M. hyopneumoniae* during infection is not understood.

#### Genes Related to Alternative Carbon Metabolism Were Down-Regulated During Infection in *Mycoplasma hyopneumoniae*

There was down-regulation under *in vivo* conditions of the glycerol uptake facilitator and the glycerol kinase (Figure 4, homologs of MHP_RS01925 and MHP_RS01930). Glycerol is a possible energy source for *M. hyopneumoniae* and is used for production of cardiolipin (Kamminga et al., 2017b). Glycerol consumption by mycoplasma species has been associated with virulence because hydrogen peroxide is released when glycerol-3-phosphate is converted to di-hydroxyacetone phosphate via the glycerol oxidase (Vilei and Frey, 2001). There was also down-regulation *in vivo* of the operons related to myo-inositol and ribose transport and catabolism (Figure 4). As the *in vitro* medium does not contain high levels of unbound myo-inositol (Kamminga et al., 2017b), up-regulation of this pathway under *in vitro* conditions can be expected. Another down-regulated operon is involved in oligopeptide transport. This could indicate that oligopeptides, which are present in a high concentration in the complex growth medium, are absent or present at lower levels in the natural niche. Transport of single amino acids could alternatively provide *M. hyopneumoniae* with amino acids needed for protein synthesis.

#### Bacterial ncRNA’s Were Differentially Expressed During Infection

Based on a best-blast hit with the strain 98 genome, we identified 606 out of 629 ncRNAs annotated in strain 232 (Lloréns-Rico et al., 2016). After filtering based on read count, we found 28 up-regulated ncRNAs *in vivo*, 66 down-regulated *in vivo* and 407 not differentially expressed (Supplementary Figure S9) based on FDR < 0.01. The role of non-coding RNAs in *M. hyopneumoniae* is not known. Given the high AT-content of the genome it is expected that most antisense RNA’s found in our datasets were the result of spurious transcription (Lloréns-Rico et al., 2016). However, some might have a regulatory role and whether these are important during infection should be further established.

#### Regulation of Bacterial Immune Evasion and Tissue Disruption Mechanisms

Using BLASTP three homologs for the *Mycoplasma* Ig binding (MIB) proteins and two homologs for the *Mycoplasma* Ig protease (MIP) identified in *Mycoplasma mycoides* were identified in the strain 98 genome. One of the MIP homologs (MHP_RS03250) was downregulated *in vivo*. Other homologs (MIP: MHP_RS03200, MIB: MHP_RS02265, MHP_RS03205 and MHP_RS03245) identified in the strain 98 genome had sufficient read numbers but were not differentially expressed. IgG processing is expected to be an important mechanism to prevent detection and clearance by the host. The Friis medium used to grow the *in vitro* cultures contains commercial porcine serum which probably contains *M. hyopneumoniae* specific antibodies which presence could explain induction of MIB-MIP activity *in vitro*. Aminopeptidases, M42 glutamyl aminopeptidase (MHP_RS01270) and leucine aminopeptidase (MHP_RS02375) which bind plasminogen and facilitate activation to plasmin were not found differentially expressed *in vivo*.

### RNA Sequencing Results Do Not Agree With Microarray Data

Previously microarrays have been used to study the transcriptome of *M. hyopneumoniae* during lung infection (Madsen et al., 2008). We found no correlation (Spearman’s coefficient = -0.14, Supplementary Figure S10) between fold changes for 162 differentially expressed genes reported in this microarray study and fold changes observed in our study. This could be due to: (1) different *M. hyopneumoniae* strains were used, (2) pigs with a different genetic background and age were used, (3) different methods were used to isolate bacteria and bacterial RNA and (4) presence of host RNA could have caused non-specific binding in the microarray study. It was recently shown that differentially expressed genes at low transcript levels could be identified with RNAseq and validated with qPCR while differentially expressed genes identified with microarrays could not be validated with qPCR when transcript levels were low (Wang et al., 2014). Since the lung flush samples contained a high quantity of background RNA and low bacterial transcript levels, RNAseq seems a more suitable method to determine bacterial transcript levels in this sample type.

## Conclusion

In this study we analyzed the host and bacterial response to *Mycoplasma hyopneumoniae* lung infection using separate sample types and separate sample treatment methods. In the host transcriptomes of infected lung tissue, we found upregulation of pathways in the immune system and pathways related to responses to tissue damage. In the bacterial transcriptomes, we found up-regulated genes *in vivo* with functions related to F_1_-like ATPase, nucleotide metabolism and glycerol-3-phosphate transport while down-regulated genes were related to cilium adhesion, cell division, glycerol metabolism and alternative carbon metabolism (myo-inositol and ribose). The insight in the *in vivo* bacterial transcriptome could be used to develop alternative vaccines to protect against *M. hyopneumoniae* infection or develop improved treatment methods.

Our dataset did not contain enough detail to pinpoint *Mycoplasma hyopneumoniae* specific host-pathogen interactions. Further insights in specific host responses to infection could be obtained if more regions from the infected lung including lesions in different stages of development, specific cell types and/or artificial tissue systems are sequenced. We expect that the method for lung flush and RNA extraction we developed in this study could be widely applied to study the transcriptome of pathogens that reside at the epithelial surface of lung tissue.

## Nomenclature

Metabolite abbreviations are explained in Supplementary Table S7.

## Data Availability Statement

The datasets generated for this study can be found in the NCBI BioProject PRJNA593525.

## Ethics Statement

The animal studies were reviewed and approved by an independent ethical committee. The experiments were conducted according to EU directive 2010 63/EU and the relevant Dutch legislation (Act on Animal Experimentation, permit number AVD221002016561).

## Author Contributions

TK, JB, VM, and PS contributed to the study design. TK and NB interpreted the results. TK, NB, PS, and JB drafted the manuscript. All authors revised the manuscript, approved the final version and took responsibility for accuracy and integrity of the work.

## Conflict of Interest

TK and JB were employed by MSD Animal Health, a pharmaceutical company producing veterinary vaccines. The remaining authors declare that the research was conducted in the absence of any commercial or financial relationships that could be construed as a potential conflict of interest.

## Footnotes

1 ftp://ftp.ensembl.org/pub/release-92/fasta/sus_scrofa/cdna/
