# Supplementary material for: Combined Transcriptome Sequencing of Mycoplasma hyopneumoniae and Infected Pig Lung Tissue Reveals Up-Regulation of Bacterial F1-Like ATPase and Down-Regulation of the P102 Cilium Adhesin in vivo
Source: Front Microbiol. 2020 Jul 20;11:1679. doi: 10.3389/fmicb.2020.01679 (PMC7379848; doi:10.3389/fmicb.2020.01679)
Supplement: Supplementary file 2 [file Data_Sheet_2.docx]

Supplementary Material

Combined transcriptome sequencing of *Mycoplasma hyopneumoniae* and infected pig lung tissue reveals up-regulation of bacterial F1-like ATPase and down-regulation of the P102 cilium adhesin *in vivo*

Authors: Tjerko Kamminga^1,2^, Nirupama Benis^1^, Vitor Martins dos Santos^1^, Jetta J.E. Bijlsma^3^, Peter J. Schaap^1*^

^1^ Laboratory of Systems and Synthetic Biology, department of Agrotechnology and Food Sciences, Wageningen University and Research, Wageningen, The Netherlands; ^2^ Bioprocess Technology and Support, MSD Animal Health, Boxmeer, The Netherlands; ^3^ Discovery & Technology, MSD Animal Health, Boxmeer, The Netherlands

*Corresponding author: [peter.schaap@wur.nl](mailto:peter.schaap@wur.nl)


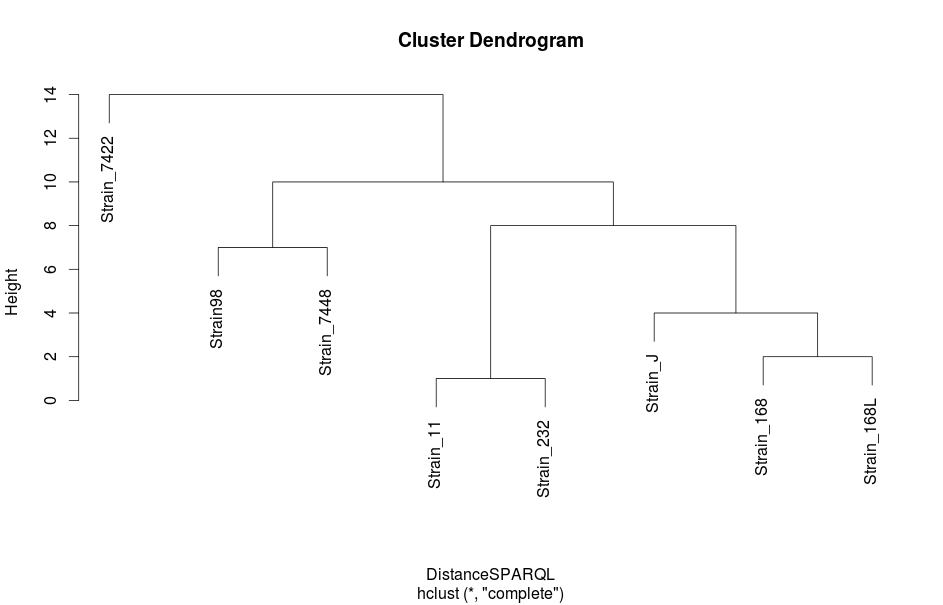


Figure S1: functional clustering based on Manhattan distance calculated from the presence/absence matrix of the *M. hyopneumoniae* accessory domainome of multiple strains


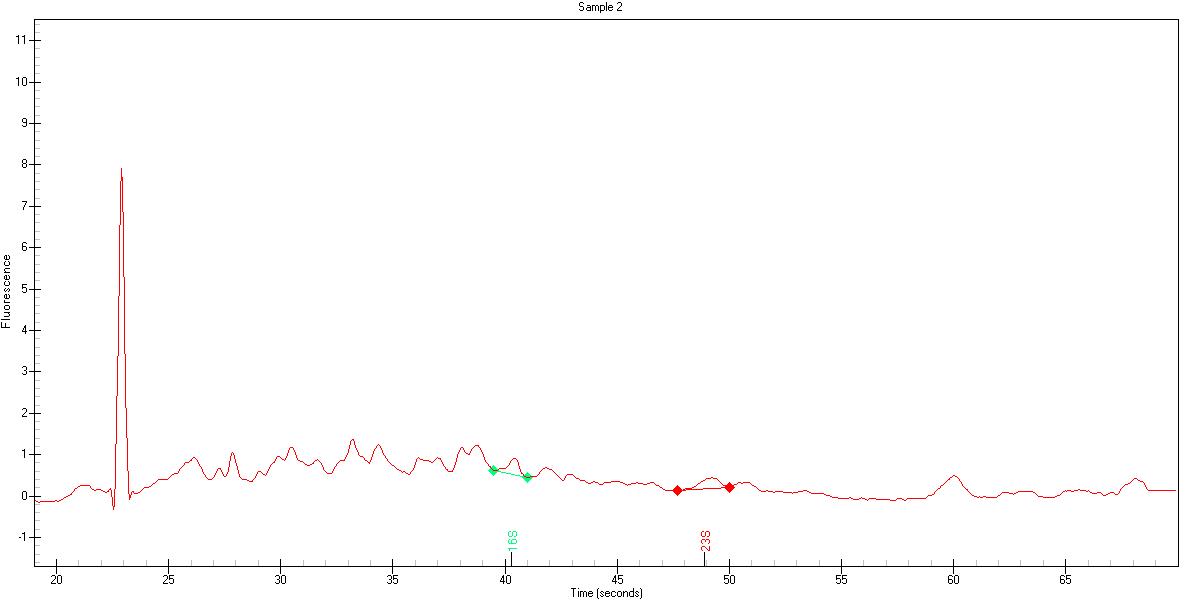


Figure S2: Experion profile sample F1-E


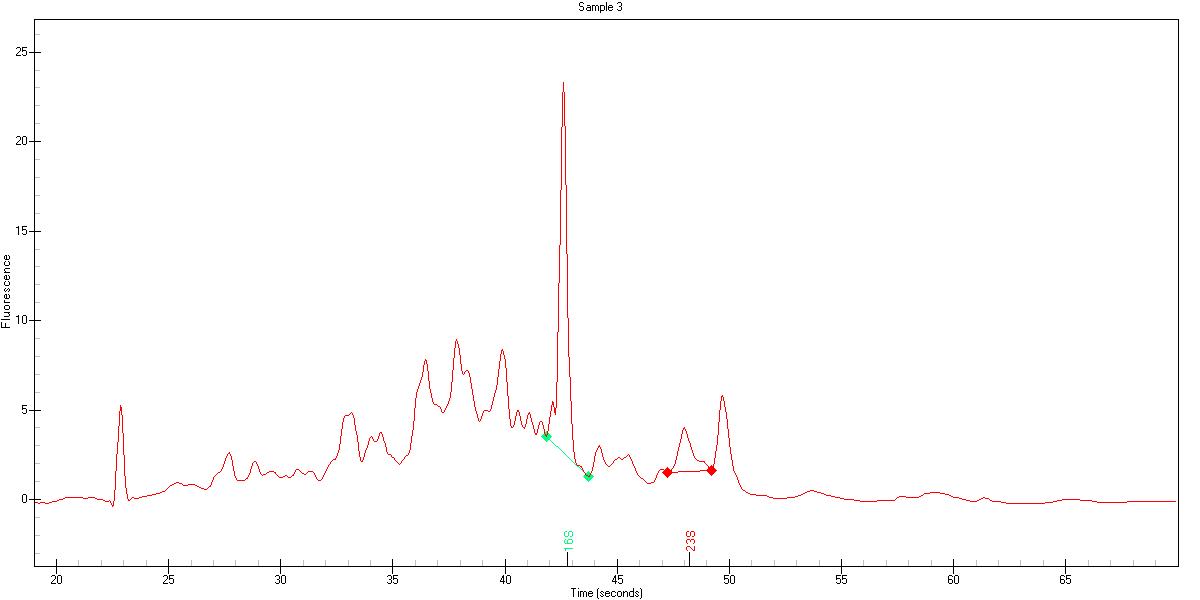


Figure S3: Experion profile sample F1-NE


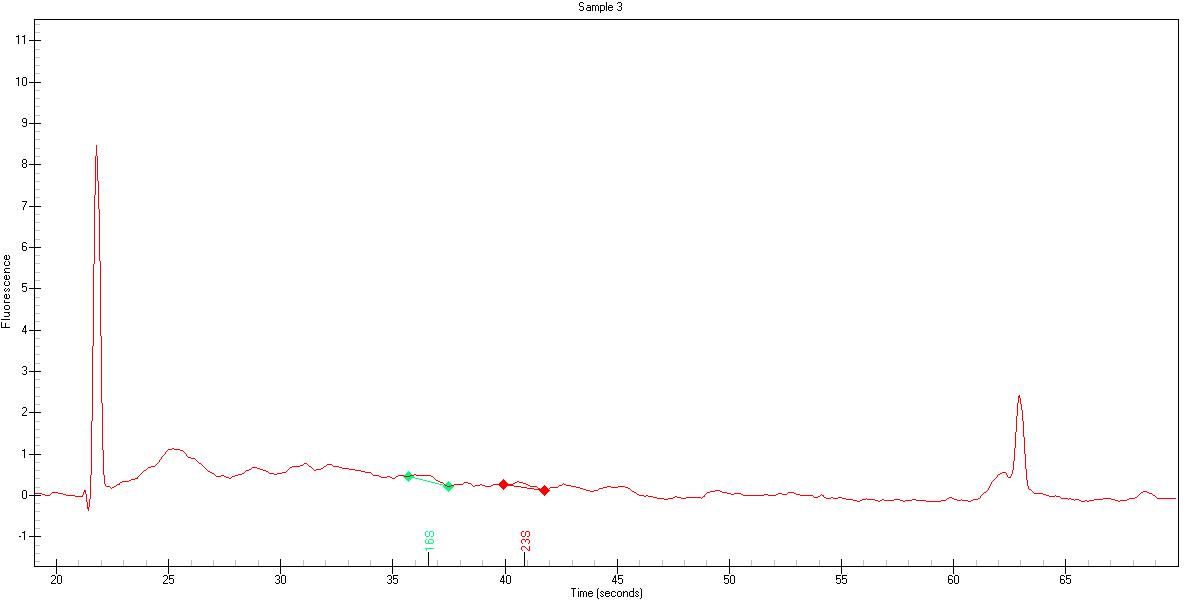


Figure S4: Experion profile sample F2-E


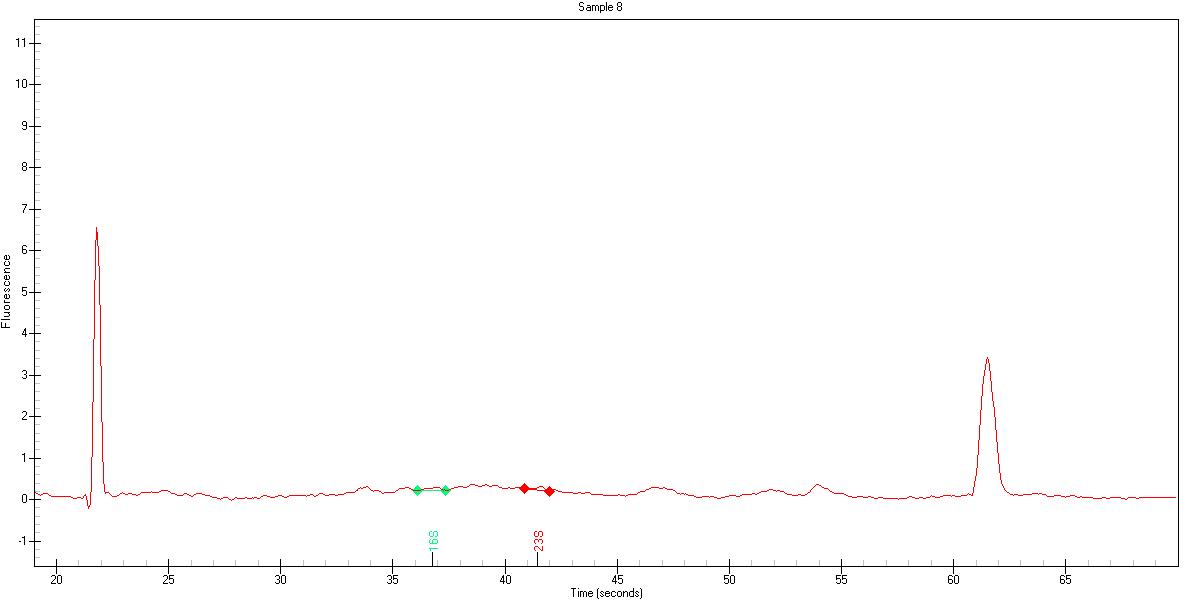


Figure S5: Experion profile sample F3-E


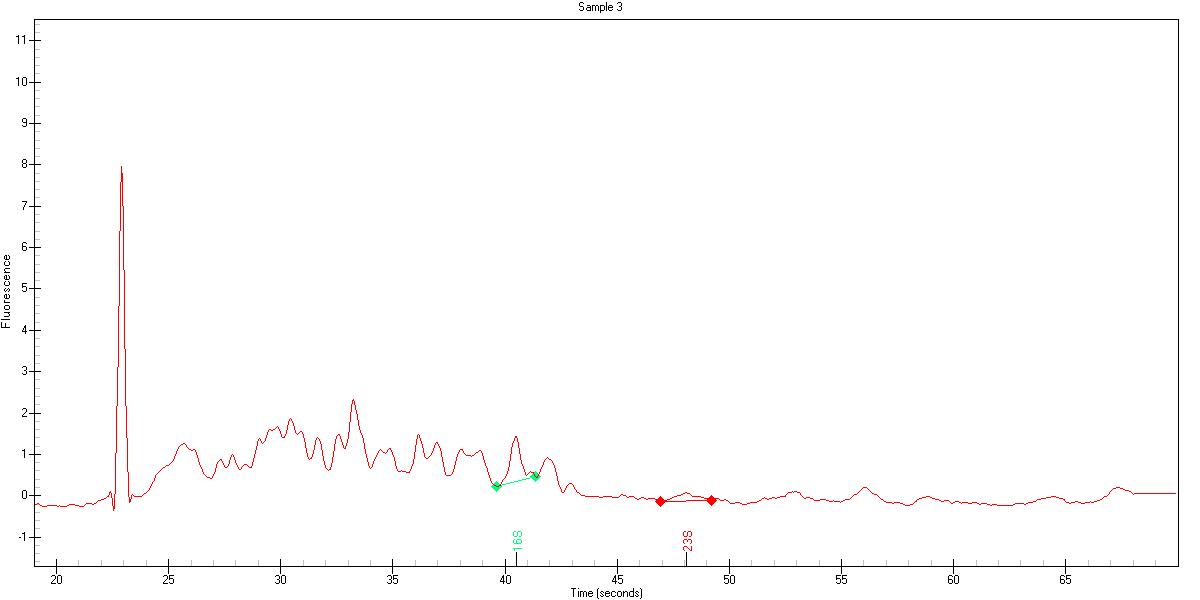


Figure S6: Experion profile sample F4-E


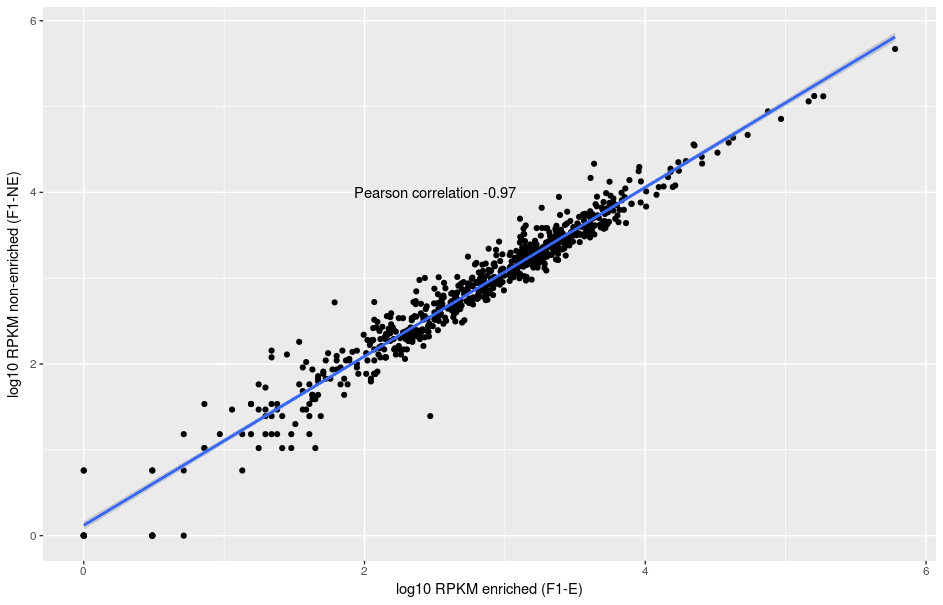


Figure S7: correlation analysis between expression levels of genes (log 10 RPKM) in an enriched flush sample (F1-E) and a non-enriched flush sample (F1_NE) sample, the same flush sample was used as input for RNA purification and sequencing, all steps in the sample treatment were followed.


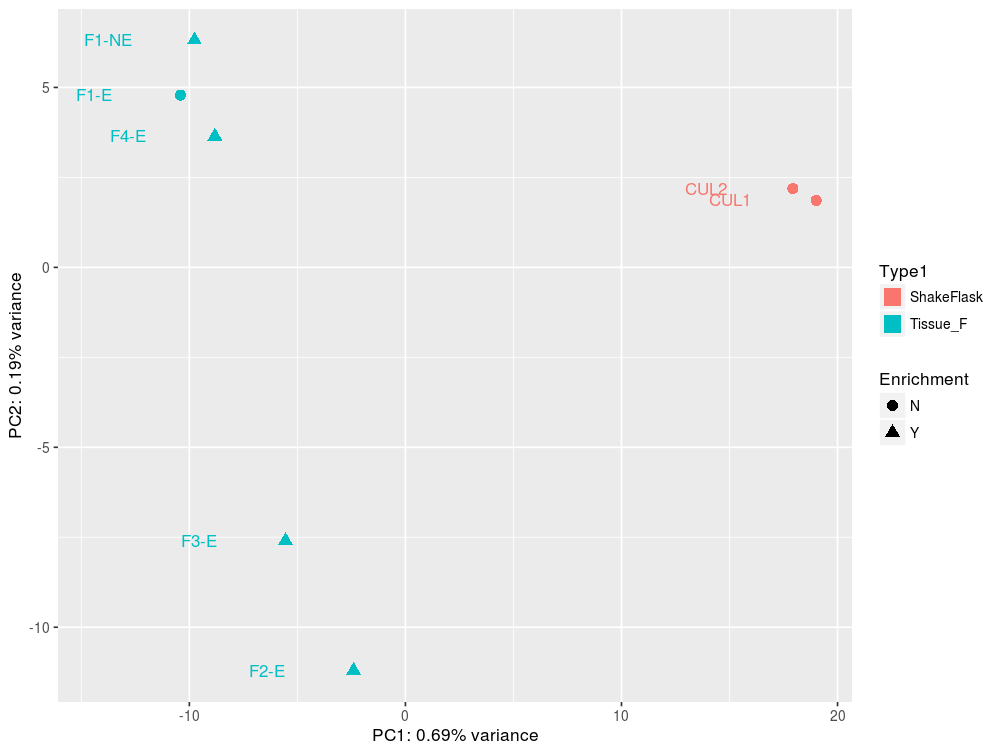


Figure S8: PCA analysis (selected flush samples, tissue and culture samples).


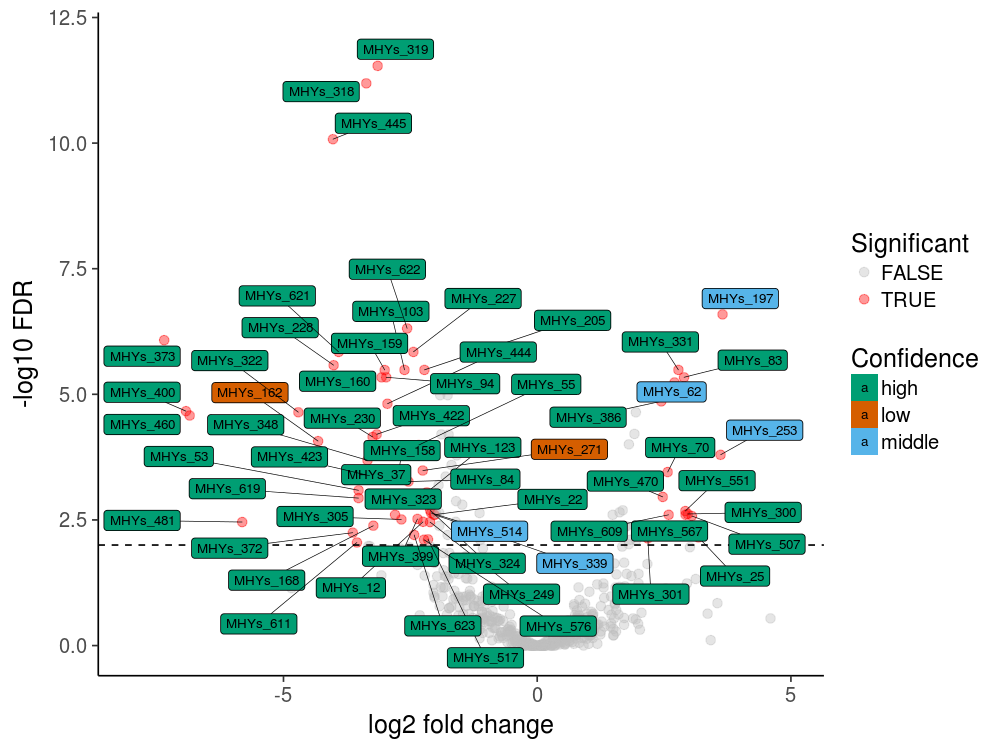


Figure S9 Volcano plot showing differentially expressed non-coding RNAs. Significance was determined based on fold change (>2 log2 up- or downregulated) and FDR<0.01. Confidence score is indicated and is based on blast data output (bit-score divided by query length: <1=low, 1-1.5=middle and >1.5=high)


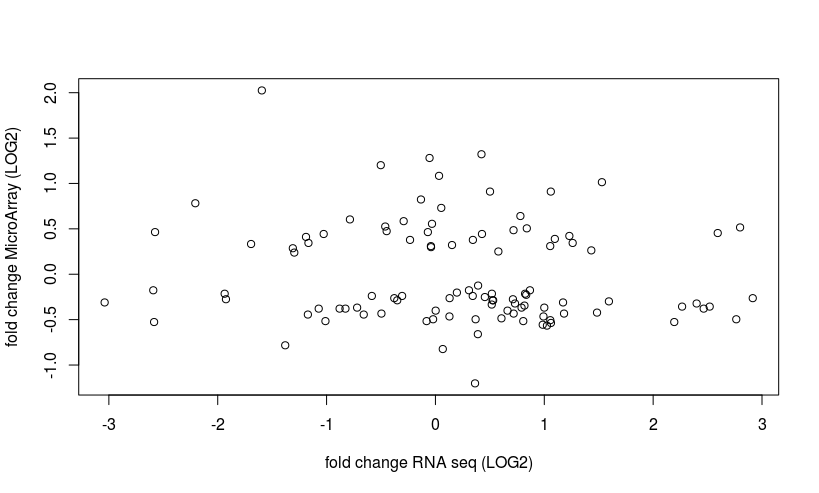


Figure S10 correlation between log2 fold changes reported for DE genes by Madsen *et al* (Madsen et al., 2008) and the same genes in our study.

**References**

Madsen ML, Puttamreddy S, Thacker EL, Carruthers MD, Minion FC. 2008. Transcriptome changes in Mycoplasma hyopneumoniae during infection. *Infect. Immun.* **76**:658–63.
